# Comparative morphological studies on the carcinogenic effect of 7,12-dimethylbenz(A)anthracene (DMBA) in normal or intrasplenic ovarian tissue of C3H mice.

**DOI:** 10.1038/bjc.1975.265

**Published:** 1975-11

**Authors:** J. Hilfrich

## Abstract

**Images:**


*Br. J. Cancer* (1975) **32**, 588

### COMPARATIVE MORPHOLOGICAL STUDIES ON THE CARCINOGENIC EFFECT OF 7,12-DIMETHYLBENZ(A)ANTHRACENE (DMBA) IN NORMAL OR INTRASPLENIC OVARIAN TISSUE OF C3H MICE*

J. HILFRICH

*From the Abteilung für Experimentelle Pathologie, Medizinische Hochschule Hannover, 3000 Hannover-Kleefeld, Karl-Wiechert Allee 9, FRG*

Received 9 June 1975. Accepted 30 July 1975

**Summary.—A single intravenous injection of 100 mg/kg body weight (b.w.) of 7, 12 dimethylbenz(a)anthracene (DMBA) induces a high percentage of ovarian granulosa cell tumours in C3H mice. After implantation of ovarian tissue into the spleen of gonadectomized female C3H mice similar tumours were found, resulting from an over-stimulation by pituitary gonadotrophins. In the present study the tumour development in intrasplenic ovarian tissue was observed after an additional single intravenous application of 100 mg/kg b.w. DMBA. It was found that the induction of granulosa cell tumours did not seem to be affected by the carcinogen injection whether 12 weeks before or 12 weeks after ovarian tissue was implanted into the spleen. The morphology of these neoplasms corresponds to the DMBA induced granulosa cell tumours in orthotopic ovaries. A direct carcinogenic effect of DMBA on ovarian cells in mice could not be demonstrated but there are indications that the additional DMBA application accelerated the destruction of the oocytes, which might result in a more rapid intrasplenic tumour induction.**

OVARIAN tissue was found to develop benign granulosa or granulosa-theca cell tumours after implantation into the spleen of gonadectomized rats (Biskind and Biskind, 1944). This is due to an uninhibited stimulation of pituitary gland gonadotrophins (Heller and Jungck, 1947; Miller and Pfeiffer, 1950; Achilles and Sturgis, 1951; Lipschutz, Cerisola and Panasevich, 1964) because steroids secreted from the implant pass directly through the portal system to the liver where they are inactivated (Golden and Sevringhaus, 1938; Lipschutz *et al.*, 1964; Leavitt, Carlson and Meyer, 1971) and therefore the feedback mechanism between pituitary gland and ovarian tissue is interrupted.

Preliminary investigations (Hilfrich and Mohr, 1973; Hilfrich, 1973, 1974) have shown that in rats normal, non-implanted ovarian tissue is not affected by 7, 12 dimethylbenz(a)anthracene (DMBA) application but that implantation of ovarian tissue into the spleen, followed by intravenous DMBA treatment, resulted in the transformation from benign to malignant granulosa, a few cases of theca cell tumours, and a single androblastoma-like neoplasm. It has been suggested that the change in hormonal balance through the implantation of ovarian tissue presupposes the necessary conditions for the carcinogenic effect of DMBA.

In contrast to rats, normal mice developed granulosa cell tumours of the ovary after the administration of DMBA (Howell, Marchant and Orr, 1954; Marchant, 1957; Mody, 1960; Biancifiori, Bonser and Caschera, 1961; Kuwahara, 1967; Krarup, 1970*a*).

As gonadectomy with implantation of ovarian tissue into the spleen of female mice also leads to the development of granulosa cell tumours (Furth and Sobel,

* Supported by Deutsche Forschungsgemeinschaft.

1947; Gardner, 1955; Guthrie, 1957), the present study was undertaken to compare the influence of DMBA on tumour development in implanted ovarian tissue in mice with that in rats.

### MATERIALS AND METHODS

Ninety female, 3-month old C3H mice (Laboratory Animals Breeding and Research Centre, Bomholtgård, Denmark) of 25–30 g body weight (b.w.) were kept in groups of 3 in Makrolon cages Type II (E. Becker & Co., GMBH, Castrop-Rauxel, FRG) under standard laboratory conditions (room temperature 22 ± 2°C; relative humidity 55 ± 5%; air exchange 8 times/h), with Hope Farms RMH–TMB pelleted diet (Woerden, The Netherlands) and water *ad libitum*. Sixty mice were ovariectomized under ether anaesthesia (Pronarcosi, Hoechst AG, Frankfurt, FRG), and a piece of ovary 1–2 mm in diameter was implanted into the spleen according to the method described by Biskind and Biskind (1949). The animals received a single intravenous injection of 100 mg DMBA (special 15% fat emulsion with 7,12-dimethylbenz(a)anthracene 5 mg/g; The Upjohn Company, Kalamazoo, Michigan, USA) per kg b.w. or 10 ml/kg b.w. of the solvent (intravenous fat emulsion without dextrose; The Upjohn Company, Kalamazoo, Michigan, USA).

*Treatment groups.*—These consisted of (1) 10 mice given 10 ml solvent/kg b.w. (controls); (2) 20 mice given 100 mg DMBA/kg b.w.; (3) 10 mice, 12 weeks *after* implantation of ovarian tissue into the spleen, given 10 ml solvent/kg b.w. (controls); (4) 30 mice given 100 mg DMBA/kg b.w., 12 weeks *before* implantation of ovarian tissue into the spleen; (5) 20 mice, 12 weeks *after* implantation of ovarian tissue into the spleen, given 100 mg DMBA/kg b.w.

Dead animals were autopsied and all organs fixed in 4% buffered formalin; paraplast sections were stained with haematoxylin and eosin and van Gieson stain; the ovarian tumours were also stained with PAS and Alcian blue, Gomori, Masson-Goldner and Sudan III. The effective number of animals is based on the number of mice surviving after the first tumour of any site had been observed. A few mice were lost through cannibalism or the adverse effects of the surgical operation.

For statistical comparison of intrasplenic ovarian tumour incidence, the chi-square test, and for the average survival times, the U-test after Mann and Whitney (1947) were performed.

### RESULTS

Table I summarizes the average survival rates as well as the incidence of ovarian and other tumours in Groups 1 and 2 (orthotopic ovaries). In control animals no ovarian tumours were found; however, after treatment with a single dose of DMBA 78·9% of the mice showed an ovarian tumour, in one animal bilaterally (Fig. 1). The first neoplasm of the ovary was observed 29 weeks after DMBA application. Macroscopically,

TABLE I.—*Ovarian and Other Tumour Incidence in Female C3H Mice after DMBA Treatment*

| Treatment groups | No. of animals initial/effect. | Average survival after treatment in weeks (range) | Animals with: Ovarian tumours (%) | Animals with: Tumours of other sites (no.) | Animals with: Leukaemias |
|---|---|---|---|---|---|
| 1 (control) | 10/10 | 81·9 (52–123) | — | Malignant lymphoma (1) | — |
| 2 (DMBA) | 20/19 | 55·5 (27–84) | 15 (78·9) | Angiosarcoma (2) and stromal sarcoma (2) of uterus<br>Papilloma or squamous cell carcinoma of forestomach (3)<br>Fibrovascular polyp (1) and squamous cell carcinoma of vagina (2)<br>Adenoma of lung (2)<br>Adenocarcinoma of mammary gland (1) | 11 |

FIG. 1.—Bilateral granulosa cell tumour 81 weeks after DMBA treatment. ×2.

FIG. 2.—Intrasplenic granulosa cell tumour with grey-white tumour masses, haemorrhages and necroses, 99 weeks after DMBA treatment and 87 weeks after implantation of ovarian tissue into the spleen. ×2·5.

TABLE II.—*Ovarian and Other Tumour Incidence in Gonadectomized Female C3H Mice after Implantation of Ovarian Tissue into the Spleen and DMBA Treatment*

| Treatment groups | No. of animals initial/ effect. | Average survival after ovarian implant. in weeks (range) | Intrasplenic granulosa cell tumours* (%) | Animals with tumours of other sites (no.) | Leukaemias |
|---|---|---|---|---|---|
| 3 (control, intraspl. ov.) | 10/10 | 85·0 (38–124) | 5 (50·0) | Malignant lymphoma (2)<br>Carcinoma of adrenal gland cortex (2)<br>Fibrosarcoma of mediastinum (1)<br>Fibrovascular polyp of cervix (1) | |
| 4 (DMBA, intraspl. ov.) | 30/24 | 49·8 (11–92) | 8 (33·3) | Papilloma or squamous cell carcinoma of forestomach (10)<br>Adenoma or adenocarcinoma of lung (7)<br>Adenocarcinoma of mammary gland (4)<br>Adenoma or carcinoma of adrenal gland cortex (4)<br>Stromal sarcoma (1), haemangioma (1) and fibrovascular polyp (1) of uterus<br>Retroperitoneal neuroblastoma (1)<br>Keratoacanthoma of skin (1)<br>Hepatoma (1) | 9 |
| 5 (intraspl. ov., DMBA) | 20/16 | 56·4 (29–82) | 7 (43·8) | Papilloma or squamous cell carcinoma of forestomach (4)<br>Adenoma of lung (3)<br>Adenocarcinoma of mammary gland (1)<br>Malignant thymoma (1)<br>Squamous cell carcinoma of vagina (1)<br>Adenoma of adrenal gland cortex (1)<br>Hepatoma (1) | 6 |

* Pre-neoplastic stages are not included.

these neoplasms were up to 25 mm in diameter with grey–white tumour masses, and most also exhibited extensive haemorrhages and necroses. In one case lung metastases were found. In the carcinogen treated Group 2 a large number of mice with leukaemias, as well as neoplasms other than the ovary, were detected; these were mainly of the uterus, forestomach, vagina and lung (Table I).

Table II lists the average survival rates as well as the number of animals with ovarian tumours in the spleen and those with other neoplasms occurring in Groups 3, 4 and 5. All these 3 groups demonstrated neoplastic growths in the spleen that ranged in size from small nodules only a few mm in diameter to tumours of up to 40 mm. Upon cut section yellowish or predominantly grey–white tumour masses were seen, together with blood filled cysts, haemorrhages and necroses in larger neoplasms (Fig. 2). These observations were comparable with those detected in tumours of orthotopic ovaries. Liver metastases were found, one case each in Groups 3 and 4, and 2 in Group 5.

The induced ovarian tumours demonstrated similar morphological patterns, not only in orthotopic ovaries (Group 2) but also (with or without the carcinogen application) in implanted tissue (Groups 3–5). In all cases granulosa cell tumours were found, with clusters and cords or parenchymatous growth of granulosa cells; luteinized theca cells and fibre formation were present only sparsely (Fig. 3). Follicular structures of varying sizes were also seen, the larger frequently being blood filled (Fig. 4). Furthermore, tubular adenoid formations (Fig. 5) and highly

FIG. 3.—Granulosa cell tumour (orthotopic ovary) with different sized clusters of granulosa cells, separated by narrow but distinct fibrous septa. H. and E. ×300.

FIG. 4.—Granulosa cell tumour (orthotopic ovary) with follicular structures, the larger blood filled (Left). H. and E. ×140.

FIG. 5.—Tubular adenoid formations as well as luteinized stromal (theca) cells in an intrasplenic granulosa cell tumour. H. and E. ×350.

FIG. 6.—Extensive luteinized area and tubular adenoid structures as well as foci of granulosa cell proliferations originated in intrasplenic ovarian tissue. H. and E. ×300.

luteinized areas (Fig. 6) resembling (when extensive) a luteoma were detected; these morphological alterations mostly preceded the described granulosa cell proliferations. In the granulosa cell tumours numerous mitoses and infiltrative growth could be observed, in addition to necroses and haemorrhages in the more extensive neoplasms.

In the DMBA treated Groups 4 and 5 the average survival time after implantation of ovarian tissue into the spleen was significantly lower ($P < 0{\cdot}01$, $P < 0{\cdot}05$ respectively) than in the control Group 3 (Table II). Furthermore, the survival time of intrasplenic granulosa cell tumour bearing animals was significantly shortened in Groups 4 and 5 ($P = 0{\cdot}005$) compared with Group 3. However, there was no statistically significant difference ($0{\cdot}9 > P > 0{\cdot}8$) in the incidence of ovarian tumours in the spleen (Table II) among untreated mice (Group 3), the mice treated with DMBA 12 weeks before (Group 4), or 12 weeks after (Group 5) implantation of ovarian tissue into the spleen.

### DISCUSSION

The present study has shown that the development of granulosa cell tumours after implantation of ovarian tissue into the spleen of C3H mice is not affected by additional DMBA application; the morphology of these neoplasms corresponds to the DMBA induced granulosa cell tumours in orthotopic ovaries. Similar findings were described by Li, Gardner and Kaplan (1947) when they used x-ray irradiated ovaries for intrasplenic implantation. Howell *et al.* (1954) reported the induction of granulosa cell tumours in orthotopic ovaries of mice after repeated skin painting with DMBA. Marchant (1957), Mody (1960) as well as Krarup (1969, 1970*a*,*b*) could show that DMBA application to mice led to a rapid destruction mainly of the small oocytes which, therefore, seem to be the primary target cells of DMBA in the ovaries of mice. Furthermore, Krarup and Loft (1971) observed that the carcinogenic effect of DMBA on mouse ovaries is related to the immediate destruction of small oocytes, whereas the prolonged retention of DMBA after intraperitoneal injection is of no significance for neoplastic development.

Further, the induction of granulosa cell tumours in intrasplenic ovarian implants of mice (Furth and Sobel, 1947; Gardner, 1955; Guthrie, 1957) and by x-ray irradiation (Furth and Butterworth, 1936; Lick, Kirschbaum and Mixer, 1949; Kaplan, 1950; Guthrie, 1958) preceded the destruction of the oocytes. The developed ovarian tumours in all the aforementioned induction methods were similar and of granulosa cell origin. Therefore, the authors assumed that the over-stimulation by pituitary gonadotrophins might be decisive for the tumour induction, not only in intrasplenic ovarian implants but also in degeneratively changed ovaries after DMBA treatment or irradiation because these gonads lose the ability to produce sufficient steroids to control the output of gonadotrophic hormones. The retention of a normal functioning ovary (Marchant, 1960; Jull, 1969), hypophysectomy (Marchant, 1961) or the application of an antigonadotrophic serum (Ely, 1959) prevented the ovarian tumour development.

The results of the present study confirm the hormone theory of ovarian tumour induction in mice after application of DMBA; in contrast to rats, mice demonstrate no direct carcinogenic effect of DMBA on ovarian cells, even under the conditions of implantation into the spleen. Since in the DMBA treated Groups 4 and 5 the survival time of intrasplenic granulosa cell tumour bearing animals was significantly lower compared with control mice (Group 3), it is possible that the additional DMBA application accelerated the destruction of the oocytes, which might result in a more rapid intrasplenic tumour induction.

I am grateful to Christine Murphy for assisting with the manuscript.

### REFERENCES

ACHILLES, W. E. & STURGIS, S. H. (1951) The Effect of the Intrasplenic Ovarian Graft on Pituitary Gonadotropins. *Endocrinology*, **49**, 720.

BIANCIFIORI, C., BONSER, G. M. & CASCHERA, F. (1961) Ovarian and Mammary Tumours in Intact $C_3$Hb Virgin Mice Following a Limited Dose of Four Carcinogenic Chemicals. *Br. J. Cancer*, **13**, 270.

BISKIND, M. S. & BISKIND, G. R. (1944) Development of Tumours in the Rat Ovary after Transplantation into the Spleen. *Proc. Soc. exp. Biol. Med.*, **55**, 176.

BISKIND, G. R. & BISKIND, M. S. (1949) Experimental Ovarian Tumors in Rats. *Am. J. clin. Path.*, **19**, 501.

ELY, C. (1959) Inhibition of Tumor Formation in Ovarian Splenic Implants after Gonadotrophic Anti-Serum. *Cancer Res.*, **19**, 37.

FURTH, J. & BUTTERWORTH, J. S. (1936) Neoplastic Diseases Occurring among Mice Subjected to General Irradiation with X-rays. *Am. J. Cancer*, **28**, 66.

FURTH, J. & SOBEL, H. (1947) Neoplastic Transformation of Granulosa Cells in Grafts of Normal Ovaries into Spleens of Gonadectomized Mice. *J. natn. Cancer Inst.*, **8**, 7.

GARDNER, W. U. (1955) Development and Growth of Tumors in Ovaries Transplanted into the Spleen. *Cancer Res.*, **15**, 109.

GOLDEN, J. B. & SEVRINGHAUS, E. L. (1938) Inactivation of Estrogenic Hormone of the Ovary by the Liver. *Proc. Soc. exp. Biol. Med.*, **39**, 361.

GUTHRIE, M. J. (1957) Tumorigenesis in Intrasplenic Ovaries in Mice. *Cancer, N.Y.*, **10**, 190.

GUTHRIE, M. J. (1958) Tumorigenesis in Ovaries of Mice after X-irradiation. *Cancer, N.Y.*, **11**, 1226.

HELLER, C. G. & JUNGCK, E. C. (1947) Regulation of Ovarian Growth: Inhibition by Estrogen or Stimulation by Gonadotropins? *Proc. Soc. exp. Biol. Med.*, **65**, 152.

HILFRICH, J. (1973) A New Model for Inducing Malignant Ovarian Tumours in Rats. *Br. J. Cancer*, **28**, 46.

HILFRICH, J. (1974) Vergleichende experimentelle Untersuchungen zur karzinogenen Wirkung von 7, 12 Dimethylbenz(a)anthracen (DMBA) am Ovargewebe der Ratte und Maus. *Arch. Gynäk.*, in the press.

HILFRICH, J. & MOHR, U. (1973) Experiments on Malignant Transformation of Ovarian Tissue in Rats. *Proc. Fifth Quadrennial Internat. Cancer Conf.*, Perugia, Italy, in the press.

HOWELL, J. S., MARCHANT, J. & ORR, J. W. (1954) The Induction of Ovarian Tumours in Mice with 9 : 10 Dimethyl-1 : 2-Benzanthracene. *Br. J. Cancer*, **8**, 635.

JULL, J. W. (1969) Mechanism of Induction of Ovarian Tumors in the Mouse by 7, 12-Dimethylbenz(a)anthracene. VI. Effect of Normal Ovarian Tissue on Tumor Development. *J. natn. Cancer Inst.*, **42**, 967.

KAPLAN, H. S. (1950) Influence of Ovarian Function on Incidence of Radiation-induced Ovarian Tumors in mice. *J. natn. Cancer Inst.*, **11**, 125.

KRARUP, T. (1969) Oocyte Destruction and Ovarian Tumorigenesis after Direct Application of a Chemical Carcinogen (9 : 10-Dimethyl-1 : 2-Benzanthracene) to the Mouse Ovary. *Int. J. Cancer*, **4**, 61.

KRARUP, T. (1970*a*) Effect of 9, 10-Dimethyl-1,2-Benzanthracene on the Mouse Ovary. Ovarian Tumorigenesis. *Br. J. Cancer*, **24**, 168.

KRARUP, T. (1970*b*) Oocyte survival in the Mouse after Treatment with 9,10-Dimethyl-1,2-Benzanthracene. *J. Endocr.*, **46**, 483.

KRARUP, T. & LOFT, H. (1971) Presence of DMBA-$^3$H in the Mouse Ovary and its Relation to Ovarian Tumour Induction. *Acta path. microbiol. scand. Sect. A*, **79**, 139.

KUWAHARA, I. (1967) Experimental Induction of Ovarian Tumours in Mice Treated with Single Administration of 7, 12-Dimethylbenz(a)anthracene, and its Histo-pathological Observation. *Gann*, **58**, 253.

LEAVITT, W. W., CARLSON, I. H. & MEYER, R. K. (1971) Progestin Secretion and Excretion in the Ovariectomized Rat Bearing a Luteinized Ovarian Graft in the Hepatic Portal Circulation. *Endocrinology*, **88**, 16.

LI, M. H., GARDNER, W. U. & KAPLAN, H. S. (1947) Effects of X-ray Irradiation on the Development of Ovarian Tumors in Intrasplenic Grafts in Castrated Mice. *J. natn. Cancer Inst.*, **8**, 91.

LICK, L., KIRSCHBAUM, A. & MIXER, H. (1949) Mechanism of Induction of Ovarian Tumors by X-rays. *Cancer Res.*, **9**, 532.

LIPSCHUTZ, A., CERISOLA, H. & PANASEVICH, V. I. (1964) The Role of Pituitary Hormones in Ovarian Tumourigenesis. *Acta Un. Int. Cancer*, **20**, 1412.

MANN, H. B. & WHITNEY, D. R. (1947) On a Test of Whether One of Two Random Variables is Stochastically Larger than the Other. *Ann. math. Stastist.*, **18**, 50.

MARCHANT, J. (1957) The Chemical Induction of Ovarian Tumours in Mice. *Br. J. Cancer*, **11**, 452.

MARCHANT, J. (1960) The Development of Ovarian Tumours in Ovaries Grafted from Mice Pretreated with Dimethylbenzanthracene. Inhibition by the Presence of Normal Ovarian Tissue. *Br. J. Cancer*, **14**, 514.

MARCHANT, J. (1961) The Effect of Hypophysectomy on the Development of Ovarian Tumours in Mice Treated with Dimethylbenzanthracene. *Br. J. Cancer*, **15**, 821.

MILLER, O. J. & PFEIFFER, C. A. (1950) Demonstration of Increased Gonadotrophic Hormone Production in Castrated Mice with Intrasplenic Ovarian Grafts. *Proc. Soc. exp. Biol. Med.*, **75**, 178.

MODY, J. K. (1960) The Action of Four Carcinogenic Hydrocarbons on the Ovaries of IF Mice and the Histogenesis of Induced Tumours. *Br. J. Cancer*, **14**, 256.


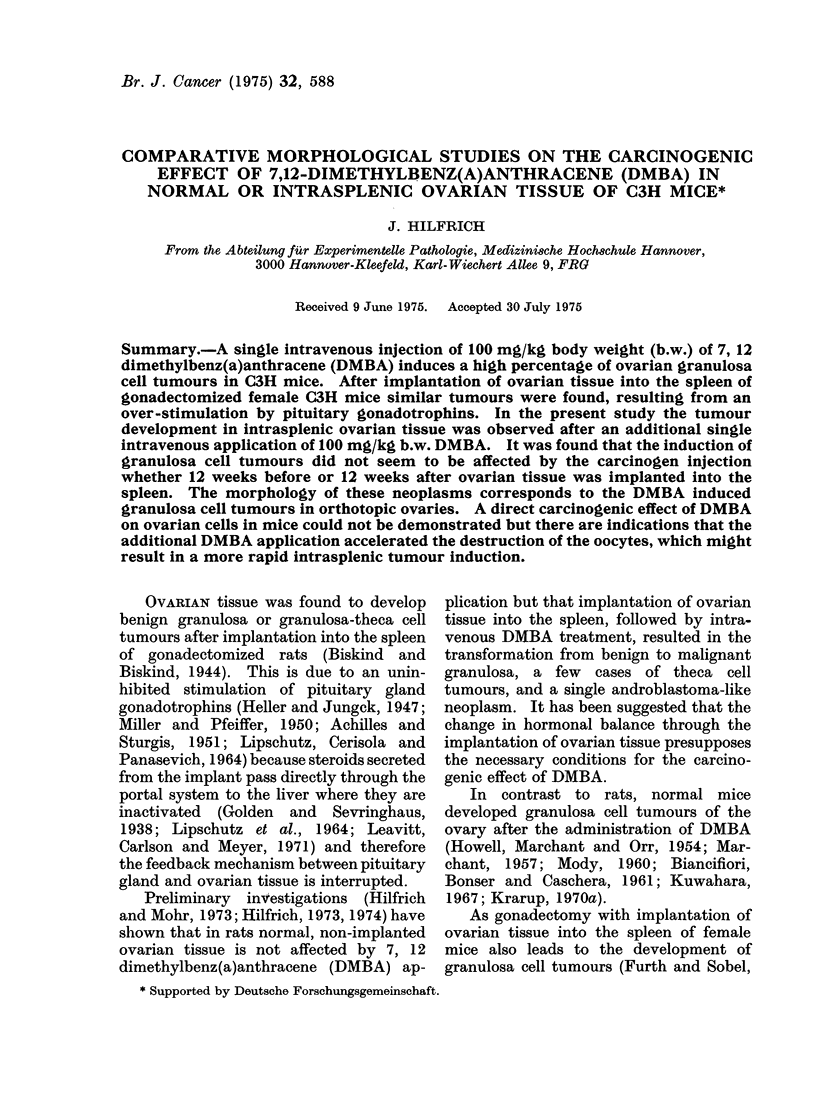

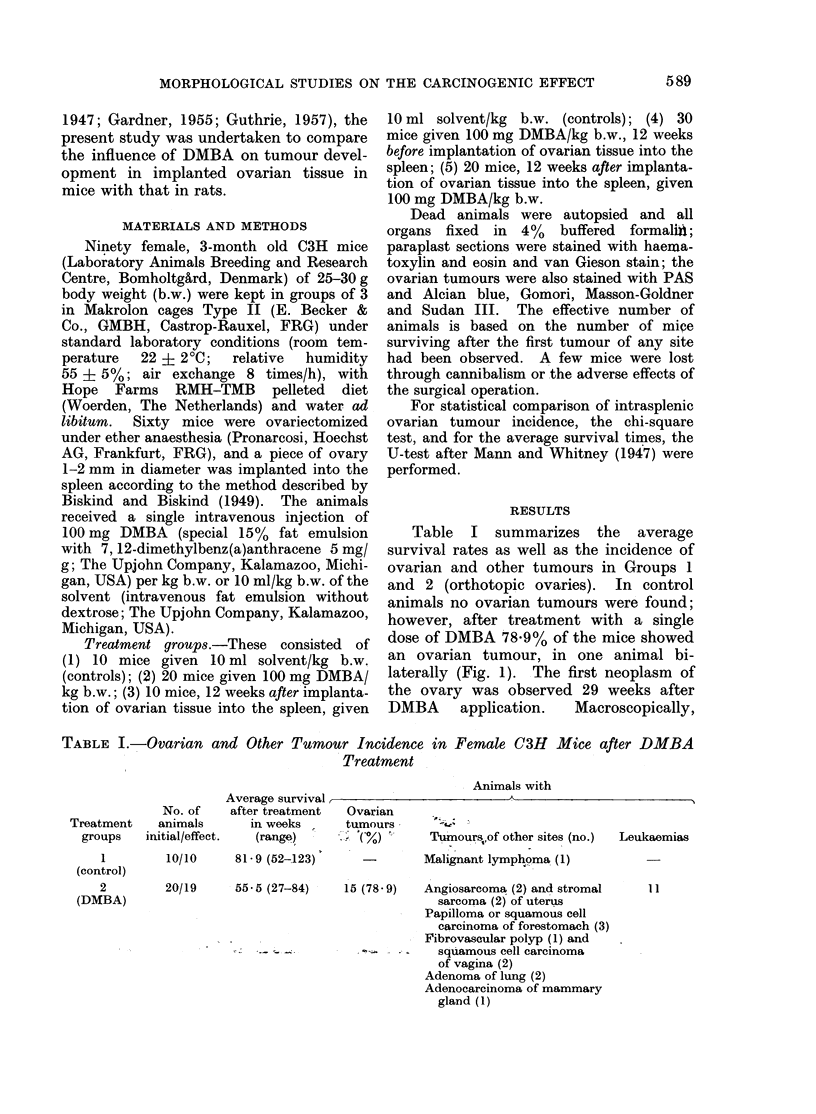

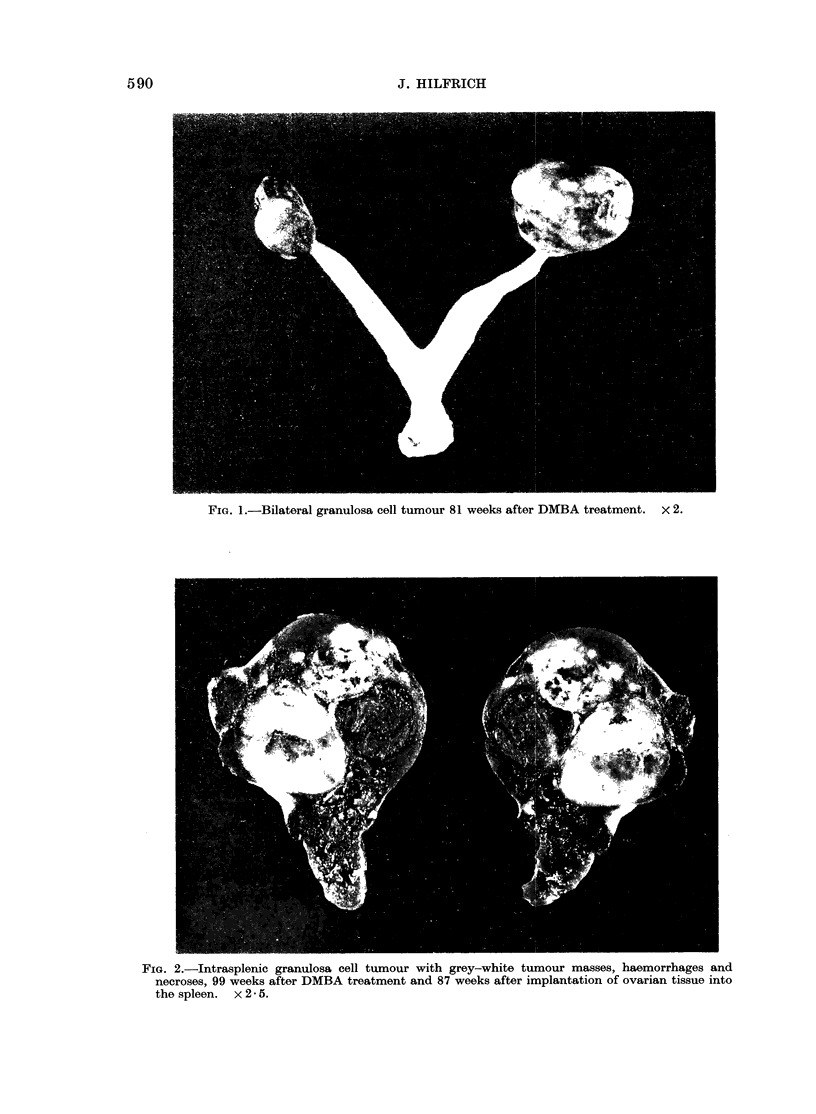

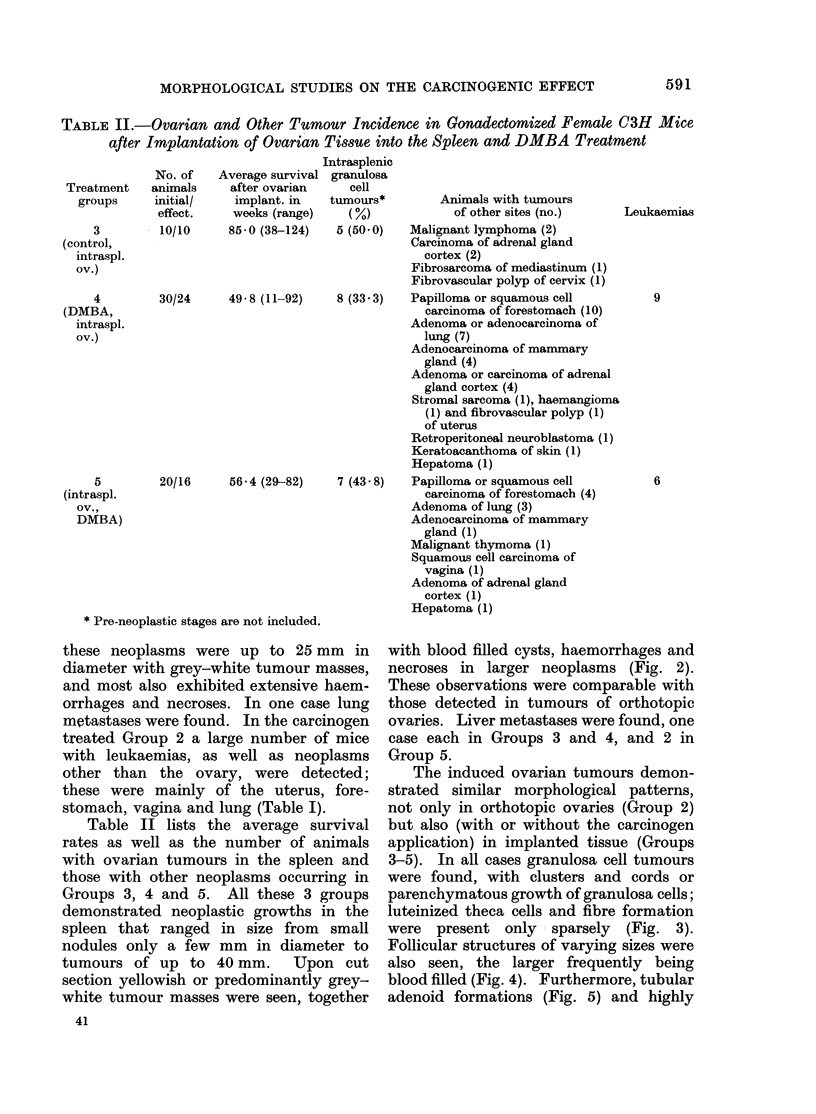

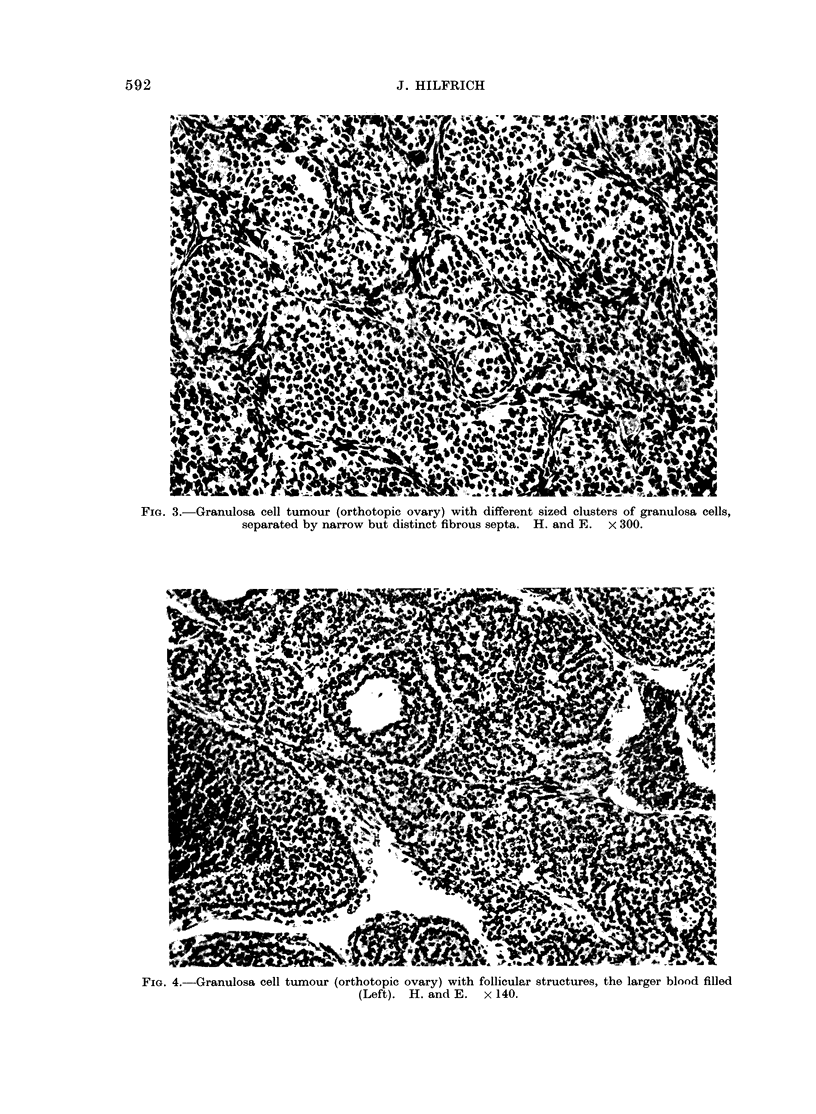

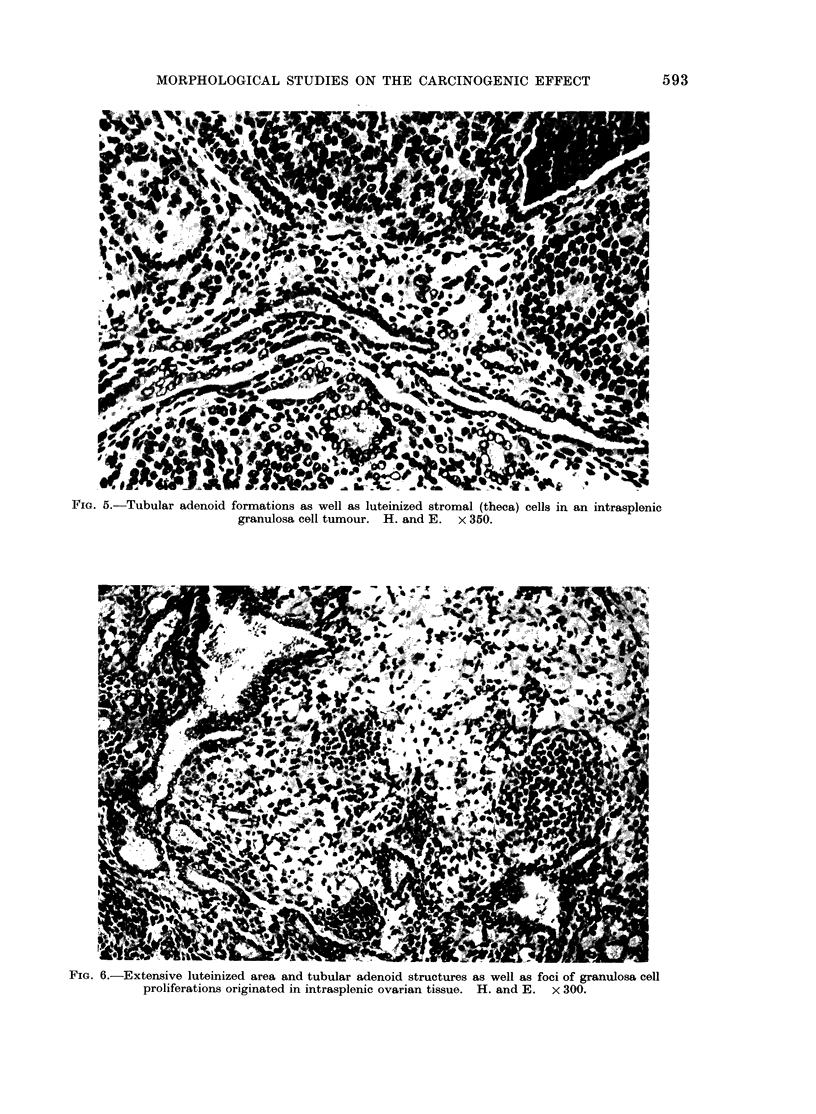

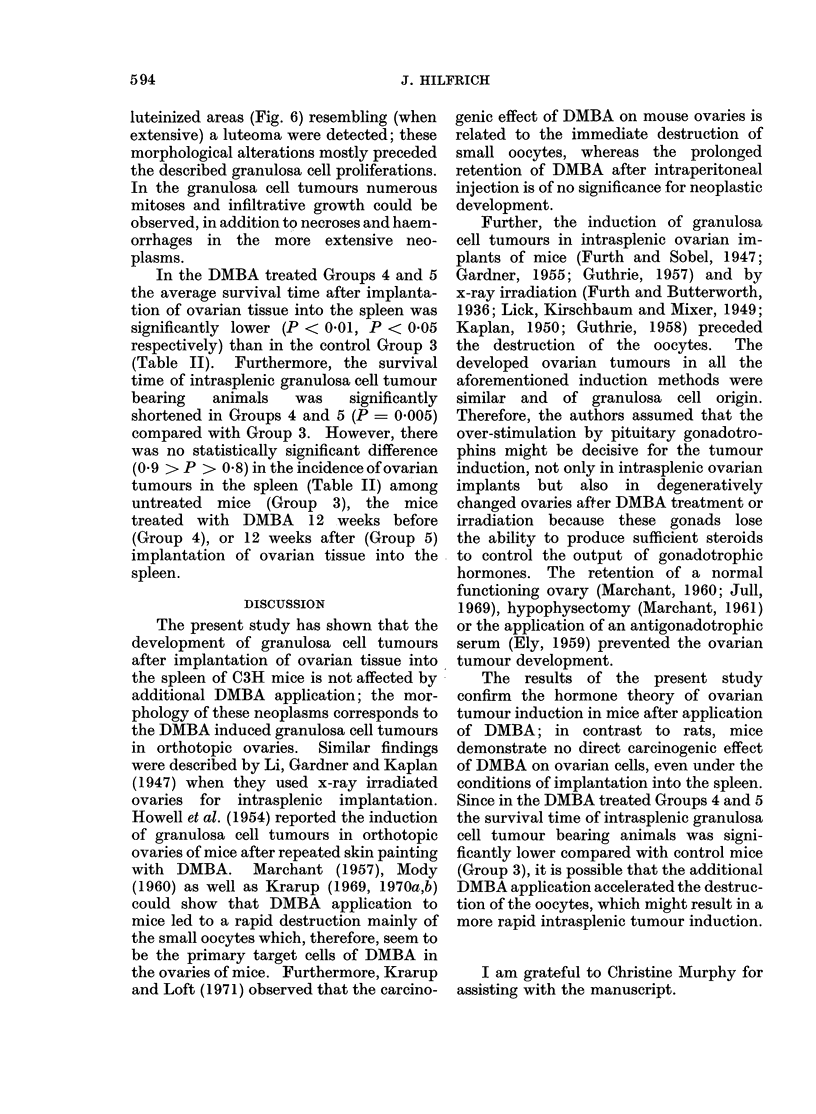

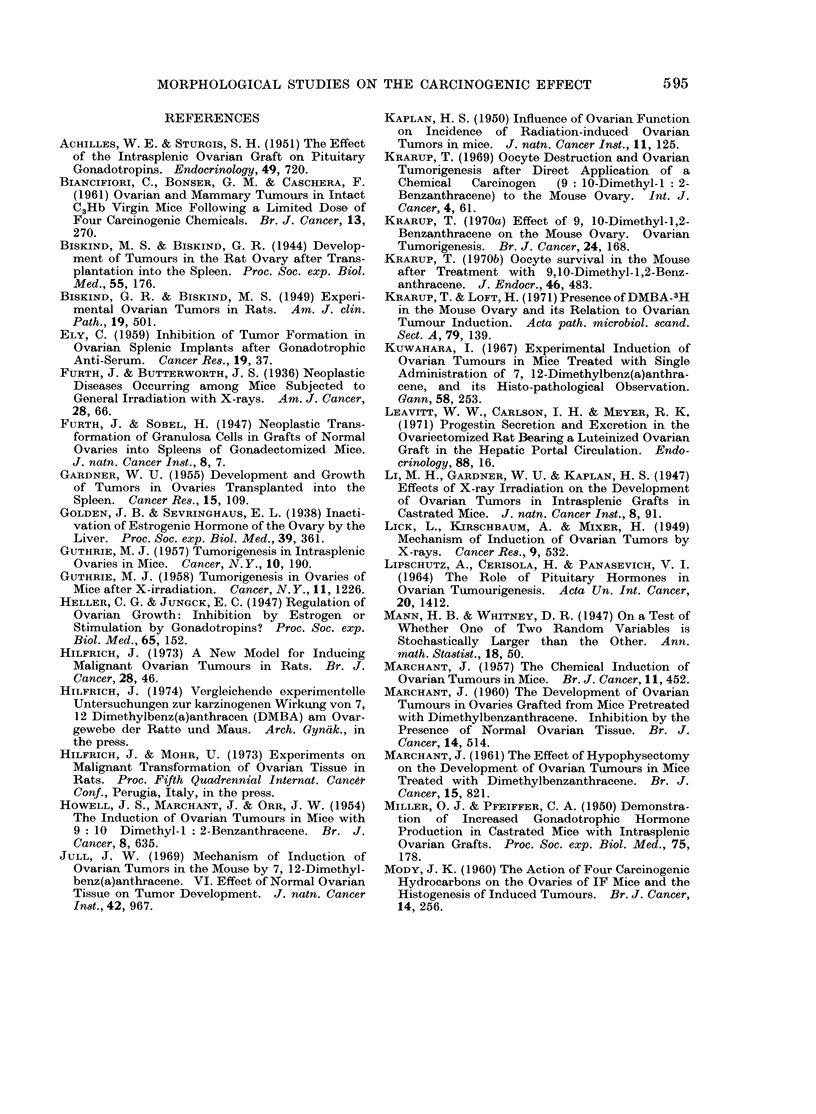


## References

[OCR_00453] ACHILLES W. E., STURGIS S. H. (1951). The effect of the intrasplenic ovarian graft on pituitary gonadotropins.. Endocrinology.

[OCR_00476] ELY C. A. (1959). Inhibition of tumor formation in ovarian splenic implants after gonadotrophic antiserum.. Cancer Res.

[OCR_00493] GARDNER W. U. (1955). Development and growth of tumors in ovaries transplanted in the spleen.. Cancer Res.

[OCR_00503] GUTHRIE M. J. (1957). Tumorigenesis in intrasplenic ovaries in mice.. Cancer.

[OCR_00534] HOWELL J. S., MARCHANT J., ORR J. W. (1954). The induction of ovarian tumours in mice with 9:10-dimethyl-1:2-benzanthracene.. Br J Cancer.

[OCR_00516] Hilfrich J. (1973). A new model for inducing malignant ovarian tumours in rats.. Br J Cancer.

[OCR_00540] Jull J. W. (1969). Mechanism of induction of ovarian tumors in the mouse by 7,12-dimethylbenz[a]anthrace. VI. Effect of normal ovarian tissue on tumor development.. J Natl Cancer Inst.

[OCR_00547] KAPLAN H. S. (1950). Influence of ovarian function on incidence of radiation-induced ovarian tumors in mice.. J Natl Cancer Inst.

[OCR_00559] Krarup T. (1970). Effect of 9,10-dimethyl-1,2-benzanthracene on the mouse ovary. Ovarian tumorigenesis.. Br J Cancer.

[OCR_00569] Krarup T., Loft H. (1971). Presence of DMBA-3H in the mouse ovary and its relation to ovarian tumour induction.. Acta Pathol Microbiol Scand A.

[OCR_00552] Krarup T. (1969). Oocyte destruction and ovarian tumorigenesis after direct application of a chemical carcinogen (9:0-dimethyl-1:2-benzanthrene) to the mouse ovary.. Int J Cancer.

[OCR_00564] Krarup T. (1970). Oocyte survival in the mouse ovary after treatment with 9,10-dimethyl-1,2-benzanthracene.. J Endocrinol.

[OCR_00575] Kuwahara I. (1967). Experimental induction of ovarian tumors in mice treated with single administration of 7,12-dimethylbenz[a]anthracene, and its histopathological observation.. Gan.

[OCR_00600] LIPSCHUTZ A., CERISOLA H., PANASEVICH V. I. (1964). THE ROLE OF PITUITARY HORMONES IN OVARIAN TUMOURIGENESIS.. Acta Unio Int Contra Cancrum.

[OCR_00582] Leavitt W. W., Carlson I. H., Meyer R. K. (1971). Progestin secretion and excretion in the ovariectomized rat bearing a luteinized ovarian graft in the hepatic portal circulation.. Endocrinology.

[OCR_00612] MARCHANT J. (1957). The chemical induction of ovarian tumours in mice.. Br J Cancer.

[OCR_00615] MARCHANT J. (1960). The development of ovarian tumours in ovaries grafted from mice pretreated with dimethylbenzanthracene. Inhibition by the presence of normal ovarian tissue.. Br J Cancer.

[OCR_00622] MARCHANT J. (1961). The effect of hypophysectomy on the development of ovarian tumours in mice treated with dimethylbenzanthracene.. Br J Cancer.

[OCR_00628] MILLER O. J., PFEIFFER C. A. (1950). Demonstration of increased gonadotrophic hormone production in castrated mice with intrasplenic ovarian grafts.. Proc Soc Exp Biol Med.

[OCR_00635] MODY J. K. (1960). The actio of four carcinogenic hydrocarbons on the ovaries of IF mice and the histogenesis of induced tumours.. Br J Cancer.

